# Supplementary material for: Imaging Cataract-Specific Peptides in Human Lenses
Source: Cells. 2022 Dec 14;11(24):4042. doi: 10.3390/cells11244042 (PMC9776990; doi:10.3390/cells11244042)
Supplement: Supplementary file 1 [file cells-11-04042-s001.zip › cells-2043940 supplementary.pdf]

Supplemental Materials

Supplemental Section 1

Sample preparation and MALDI imaging for supplemental data

20 µm thickness frozen equatorial cryostat sections were thaw-mounted on Indium-Tin-Oxide (ITO) coated slides (Delta Technologies, Loveland, CO) and vacuum desiccated for 30 min. The sections were washed 3x30 sec with 50%ACN in water as described previously [11]. Sinapinic acid (20 mg/mL) in 50:49.9:0.1 ACN:H2O:TFA was applied to tissue sections using a Portrait 630 acoustic reagent multispotter (Labcyte, Inc., Sunnyvale, CA). A total of 47 droplets of ~188 pL each were applied to each raster spot across the entire tissue with a diameter of 300 µm center-to-center spacing. Samples were then placed into in a desiccator in the dark until analysis. IMS analysis was performed in the linear positive mode on a time-of-flight mass spectrometer (Autoflex III Linear; Bruker Daltonik, Bremen, Germany) or on a Bruker SolariX 9.4T FT-ICR mass spectrometer (Bruker Daltonics, Billerica, MA, USA) equipped with a Smartbeam II 2kHz Nd:YAG (355 nm) laser. For Autoflex data, data were normalized to total ion current (TIC) and each m/z signal was plotted ±0.1% mass units. For FT data, data were normalized to root mean square (RMS) and each m/z signal was plotted ±0.001%.

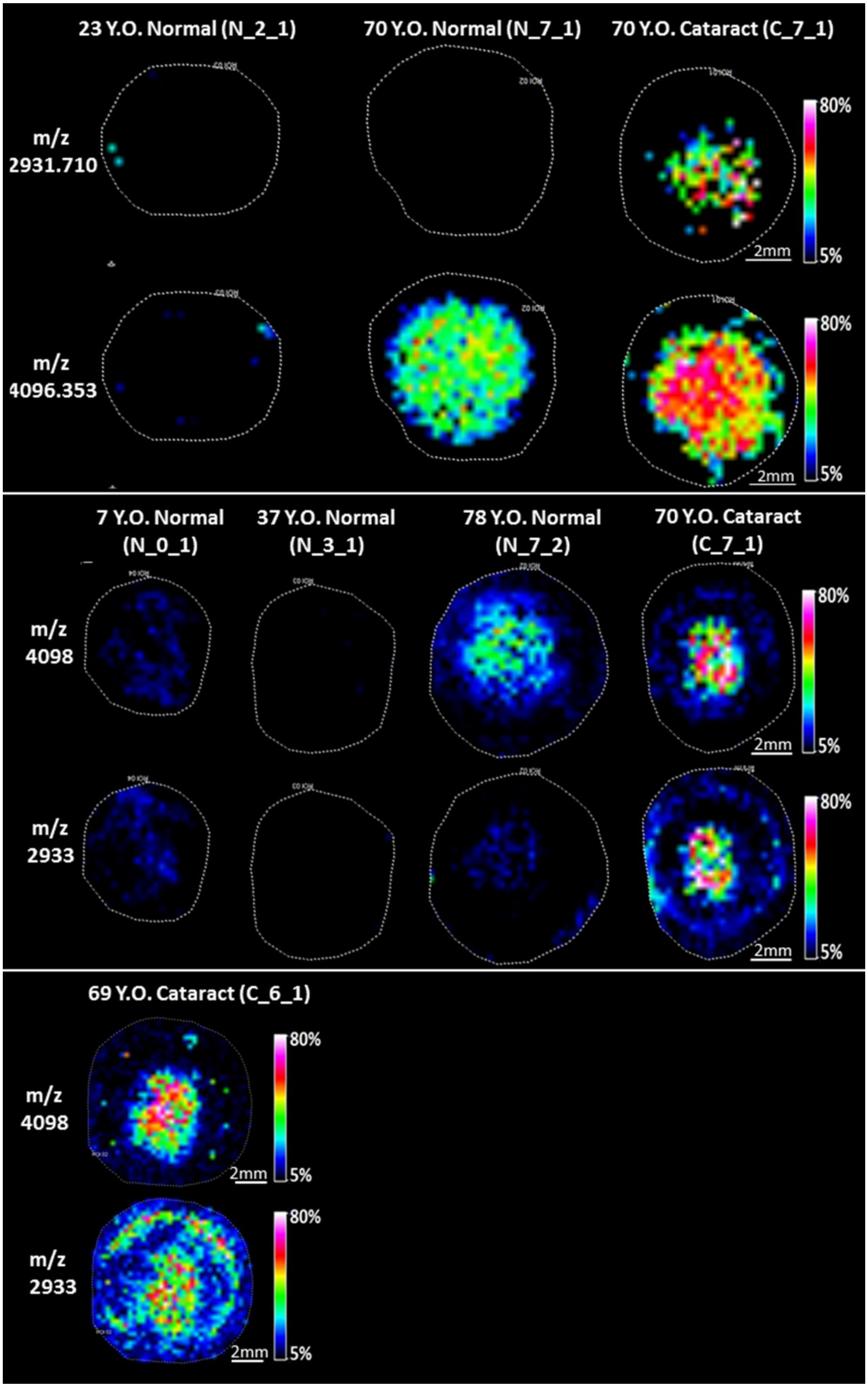
23 Y.O. Normal (N_2_1)
70 Y.O. Normal (N_7_1)
70 Y.O. Cataract (C_7_1)
m/z 2931.710
m/z 4096.353
80%
5%
2mm
7 Y.O. Normal (N_0_1)
37 Y.O. Normal (N_3_1)
78 Y.O. Normal (N_7_2)
70 Y.O. Cataract (C_7_1)
m/z 4098
m/z 2933
69 Y.O. Cataract (C_6_1)

Supplemental Figure S1: IMS analysis of γS peptides in normal and cataract lenses. Top two rows: data were acquired on an FT-ICR mass spectrometer with a 300 µm raster step size. Bottom two rows: data were acquired on Autoflex III mass spectrometer using a 300 µm raster step size.

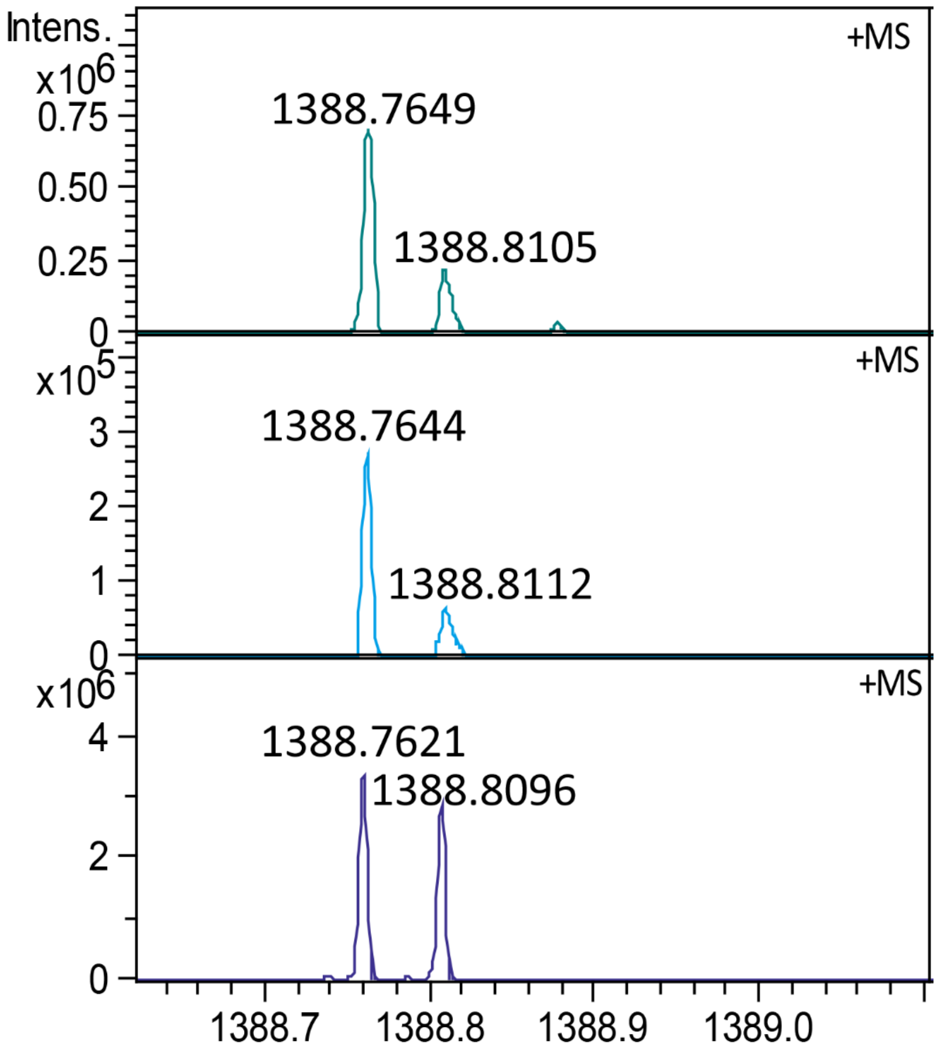

Supplemental Figure S2: The accurate mass measurement of the signal at m/z 1389 in a 54yo normal lens cortex (Top row), 55yo cataract lens cortex (middle row) and 55yo cataract lens nucleus (bottom row) on an FT-ICR mass spectrometer.
